# A Systematic Review of Physical Activity Interventions in Hispanic Adults

**DOI:** 10.1155/2012/156435

**Published:** 2012-02-08

**Authors:** Melinda J. Ickes, Manoj Sharma

**Affiliations:** ^1^Department of Kinesiology and Health Promotion, University of Kentucky, 111 Seaton Building, Lexington, KY 40506, USA; ^2^Department of Health Promotion & Education, University of Cincinnati, Teachers College 527 C, P.O. Box 210068, Cincinnati, OH 45221, USA; ^3^Department of Public Health, University of Cincinnati, Teachers College 527 C, P.O. Box 210068, Cincinnati, OH 45221, USA

## Abstract

Healthy People 2020 aims to achieve health equity, eliminate disparities, and improve the health of all groups. Regular physical activity (PA) improves overall health and fitness and has the capability to reduce risk for chronic diseases. Identifying barriers which relate to the Hispanic population is important when designing PA interventions. Therefore, the purpose was to review existing PA interventions targeting Hispanic adults published between 1988 and 2011. This paper was limited to interventions which included more than 35% Hispanic adults (*n* = 20). Most of the interventions were community based (*n* = 16), although clinical, family-based, and faith-based settings were also represented. Interventions incorporated theory (*n* = 16), with social cognitive theory and transtheoretical model being used most frequently. Social support was integral, building on the assumption that it is a strong motivator of PA. Each of the interventions reported success related to PA, social support, and/or BMI. Lessons learned should be incorporated into future interventions.

## 1. Introduction

In the United States, Hispanic Americans are the largest ethnic group after non-Hispanic whites. Hispanics or Latinos are persons of Cuba, Mexico, Puerto Rico, South or Central-America, or other Spanish culture or origin, regardless of race [1]. Between 2000 and 2007, this Latino population grew nearly 30%, currently representing 15.75% (48.4 million) of the U.S. population [2]. Projections estimate that by the year 2050, there will be 132.8 million Hispanic individuals, comprising over 30% of the USA. population [2]. The rapid increase in the Hispanic population throughout the USA. requires an understanding of Hispanic culture, health care needs, and methods to approaching health promotion [3].

One of the overarching goals of Healthy People 2020 is to achieve health equity, eliminate disparities, and improve the health of all groups [4]. In order for this to occur, it is necessary to look at the underlying factors which contribute to such disparities. Unfortunately, within the Hispanic subgroup of the USA. population, health disparities are prevalent. It is known that obesity is a significant public health concern across the USA. In fact, 68% of the adult population was classified as overweight (BMI > 25 or higher), and 33.8% classified as obese (BMI > 30 or higher) in 2007-2008 [5]. Yet, rates of overweight and obesity are significantly higher among Latinos/Mexican Americans. In 2009, Hispanic Americans were 1.2 times as likely to be obese than non-Hispanic whites [6]. In Latino women, in particular, rates have increased at alarming rates, with 73% were considered overweight or obese, compared to 61.6% of the general female population [1].

Consequences related to overweight and obesity have been cited time and time again and have been associated with an increased risk of coronary heart disease, type 2 diabetes, certain types of cancer, hypertension, dyslipidemia, stroke, liver disease, gallbladder disease, sleep apnea, respiratory problems, and osteoarthritis [7]. Unfortunately disparities related to some of these chronic diseases in Latino communities are very prevalent. Coronary heart disease is the leading cause of death in Latinos, much like the general population; however, the death rates are higher than the national average [8, 9]. In Hispanic women, coronary heart disease mortality was 40% greater than non-Hispanic whites [10]. Rates of diabetes are 2 to 3 times greater compared to non-Hispanic whites [11]. Compared to non-Hispanic whites, Hispanics in the USA. are more likely to die from diabetes (41%), stroke (18%), and chronic liver disease (62%) [6]. Implications regarding the high incidence of morbidity and mortality among the Hispanic population lend to the importance of addressing such disparities.

Regular physical activity has benefits to improve overall health and fitness and the capability to reduce risk for many of the above-mentioned chronic diseases. According to the *2008 Physical Activity Guidelines for Americans*, physical activity is anything that gets your body moving. For adults, it is recommended that both aerobic and muscle-strengthening activities be included each week: 150 minutes of moderate-intensity aerobic activity and muscle-strengthening activities on two or more days a week that work all major muscle groups [12].

Unfortunately, the proportion of the USA. population that is participating in physical activity is dismal in comparison to the known benefits. In 2008, 32.6% of the population reported no leisure-time physical activity, and only 18.2% of adults met the physical activity guidelines for Americans [4]. *Healthy People 2020 *has indicated lack of physical activity as a priority area for our nation, including objectives to reduce the proportion of adults who engage in no leisure-time physical activity and to increase those adults who are meeting the recommended levels of physical activity [4].

In the Latino population, physical inactivity appears to be more prevalent when comparing among Hispanics (37%), blacks (33%) and whites (22%) [6]. According to Neighbors et al. [13], although all Hispanic groups were less active than were non-Hispanic white individuals; much variability was seen across the subgroups: Cuban and Dominican participants were the least active (19.8% met recommendation), whereas Mexican American participants were the most active (31.9% met recommendation). In terms of gender, women were less active than were men across all subgroups. Among Hispanic women, 74% report that they do not participate in leisure-time physical activity, and this seems to decline with age [14]. There are many factors which inhibit individuals from participating in regular physical activity.

According to *Healthy People 2020, *some of the factors negatively associated with adult physical activity include advancing age, low income, lack of time, lack of motivation, overweight or obesity, and perception of poor health [4]. With the changing demographics of the USA. population, it is necessary to look at specific factors which relate to the growing Hispanic population. According to Crespo and colleagues, greater acculturation tends to be associated with increased physical activity among this segment of the population [14]. Research has indicated that Mexican Americans grasp the perceived benefits of physical activity, yet this does not translate to greater likelihood of participation [15].

Barriers to physical activity which have been reported by Latinos are varied, but common themes relate to time constraints and environmental access. Juarbe and colleagues questioned their Hispanic participants and found they perceived little or no time for social interactions (including physical activity) outside home and family because of their multiple role responsibilities [15]. Environmental variable significantly correlated with physical activity including perceived neighborhood safety and perceived access to facilities that enabled physical activities to occur [16].

Focusing on factors which contribute to the initiation of physical activity is necessary to truly impact this behavior among Hispanics. Key motivators for physical activity include a desire to be healthy for the family and the anticipated physical and psychological benefits [11]. Since activity preferences may vary among different cultures, culturally focused strategies have been advocated. Dancing, walking, gardening, and family-oriented activities are preferred in the Hispanic population [11, 17].

Support networks, or the extent to which a person is connected to others, are considered predictive of health behaviors. Within the Latino population, social support is described as having a friend who is supportive [18], knowing people who exercise or seeing people exercise [19], or being involved in a group exercise activity [20]. Reportedly, means of social support increase the likelihood of participation in physical activity [11, 17]. Self-efficacy, an individual's beliefs regarding their capacities to perform in a certain manner or produce certain results in their lives [21], has also been identified as a factor which contributes to the initiation and/or maintenance of physical activity among Hispanics [22, 23]. Considering the lack of physical activity among Hispanic Americans, and the associated barriers, the Institute of Medicine (IOM) has expressed an urgent need to initiate interventions which influence obesity-related behaviors among diverse ethnic groups [24].

## 2. Purpose

The goal of this systematic paper is to summarize the existing evidence related to physical activity interventions with the goal of obesity prevention in Hispanic adults, published between January 1988 and April 2011, in order to determine the benefits and limitations of specified strategies.

## 3. Methods

### 3.1. Study Abstraction

An extensive literature search was conducted independently by two researchers to collect studies for inclusion in this paper to increase the likelihood that all pertinent articles were retrieved. Searches were performed using the databases Academic Search Complete, CINAHL, ERIC, Health Source: Nursing/Academic Edition, MEDLINE, Scopus, SPORT Discus, and WOK Web of Science Citation Index Expanded. Various combinations of the following keywords were used including “Hispanic” or “Latino”; “adults”; “physical activity” or “exercise.” Limits of scholarly journals (peer reviewed) were set. Additional articles were identified by searching each included article reference section as well as those included in two related review articles: one focused on interventions in Hispanic women and girls and covered articles until 2007 [25] and another focused on Hispanic children and adults and covered articles until 2006 [26]. Any discrepancies were discussed until both authors agreed.

### 3.2. Inclusion/Exclusion Criteria

Inclusion criteria for including studies in this paper were: (1) publication in the English language, (2) conducted in the USA, (3) a primary research paper evaluating any physical activity intervention, (4) publications in peer reviewed journals between January 1988 and April 2011, and (5) the target audience for intervention was >35% Hispanics (over 18 years). Exclusion criteria were articles in languages other than English and case studies. Studies were not required to be randomized controlled trials (RCTs) because this is a relatively new area of research, and due to the relatively small amount of studies targeting this specific population, all were included as a first step in understanding the existing evidence of interventions targeting physical activity in Latinos and Hispanics.

### 3.3. Data Extraction

Data from studies were extracted independently by two researchers using a standardized form developed by the authors (available on request). It was determined that the first author would be assigned to be the data extractor who completed the data extraction form, and the second author would be the data checker who confirmed that the data on the extraction form were correct. Any disagreements were examined and the agreed final data were recorded. Extracted data included lead author, publication year, age of participants, percentage of Hispanic participants, theoretical framework used to guide intervention design and implementation, design, primary and secondary outcomes, measures used to obtain collected data, description of intervention, intervention frequency and duration, and main findings. These variables are summarized in Table 1.

### 3.4. Data Analysis

No statistical analysis or meta-analysis was conducted. Thus, the existing analysis reported in the reviewed articles was extracted and reported in a systematic format. In accordance with reporting guidelines for systematic reviews, a PRISMA checklist was used for this paper.

## 4. Results

### 4.1. Included Studies

**Figure 1:**
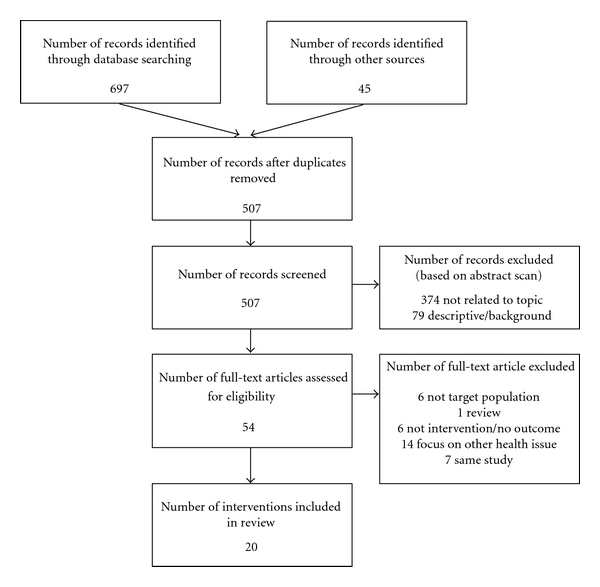
Summary of search results.

Over 700 articles were originally identified using the aforementioned search criteria. After duplicates were removed, 507 articles were screened independently by both authors. Of those, 54 full-text articles were assessed for eligibility, resulting in 20 interventions to be included in this paper. See flow diagram in Figure 1 for a summary of the systematic search. The included 20 interventions have been summarized in Table 1, giving a description of the target population, measures used, the intervention and design, and salient findings. The interventions have been arranged alphabetically by first author's last name.

### 4.2. Design and Sample

This paper was limited to interventions in which the population included greater than or equal to 35% Hispanic adults. Nine of the interventions included a 100% Hispanic population [3, 18, 27–33]; while the others ranged from 70–80% Hispanics (*n* = 6) [34–39] and 40–50% (*n* = 4) [28, 40–42]. The age of participants in the interventions ranged from 18 to 95 years, although 85% (*n* = 17) targeted middle-aged adults. Half of the interventions (*n* = 10) specifically targeted females [18, 28, 30, 32–35, 38, 39, 42], which may reflect the increased prevalence of obesity among Hispanic women [6]. In addition, several of the interventions recruited specific populations including low income (*n* = 6) [3, 18, 30, 32, 35, 43], sedentary (*n* = 4) [18, 28, 32, 40], obese (*n* = 3) [28, 32, 39], those with diabetes (*n* = 3) [3, 27, 29], and individuals at risk for cardiovascular disease (*n* = 1) [33].

Considering the design of the studies reported, 65% (*n* = 13) were randomized controlled trials [18, 25, 28, 30, 32–36, 39, 41, 44, 45], in which participants were randomly assigned to the intervention or control group. Two of the interventions were quasiexperimental [40, 42], which did not randomize the participants, yet still had a control or comparison group. A nonexperimental design was also used in four of the interventions [3, 31, 37, 38], in which control and/or comparison groups were not delineated. In addition, one of the interventions [29] used a qualitative nonexperimental design.

The number of participants within each intervention was extremely varied. To differentiate, interventions were categorized from very small to extra large sample sizes. Three of the interventions were very small (under 20 [3, 28, 40]), six were small (20–75) [27–29, 34, 36, 45], five were medium (75–150) [30, 32, 38, 41, 42], five were large (150–300) [18, 31, 35, 39, 40], and one intervention was considered to have a very large sample size with 869 participants [33].

### 4.3. Theoretical Framework

Theory was widely incorporated into the interventions, with 75% (*n* = 15) reporting the use of some theoretical framework. social cognitive theory (*n* = 5) [30, 32, 35, 39, 42] and the transtheoretical model (*n* = 5) [32–34, 37, 40] were used most frequently. Other mentioned theories included operant learning theory [18], grounded theory [29], social science [31], social-ecological model [3], self-management model [44], and reversal theory [41]. One intervention integrated the use of multiple theories, reporting the use of both social cognitive theory and the transtheoretical model [32]. Yet, 25% of the interventions (*n* = 5) did not mention using a theoretical framework to guide the intervention [27, 28, 36, 38, 45].

### 4.4. Intervention Approach

Community-based settings were the most abundant (*n* = 14), although clinical settings (*n* = 2) [25, 28], family and home-based (*n* = 3) [30, 34, 42], and faith-based settings (*n* = 1) [36] were also represented. Duration of the interventions ranged from one to three sessions (*n* = 2) [31, 33] to twelve months (*n* = 2) [35, 39]. The duration of 90% of the interventions lasted less than one year; 1.5 to 2 months (*n* = 6) [34, 36, 41, 42, 44, 45], three to four months (*n* = 6) [3, 25, 29, 30, 37, 38], six months (*n* = 3) [18, 32, 40], and 9 months (*n* = 1) [44]. Duration within sessions also varied with 20-30-minute phone calls [42] to 90-minute educational and group-led exercise sessions [18].

 A variety of strategies were used within the design and implementation of each of the interventions. Increasing social support was a focal point in a 65% (*n* = 13) of the interventions [18, 29, 30, 32, 35–40, 42, 44, 45]. Building on this concept, 45% (*n* = 9) of the interventions used walking groups [28, 29, 35, 36, 38, 39, 42, 44, 45], 30% (*n* = 6) incorporated group aerobics/dance or structured activities [3, 18, 25, 27, 30, 40]. It was also important to use culturally appropriate activities and materials throughout the interventions, as reported in 45% (*n* = 9) of the interventions [3, 18, 33, 34, 36–40]. This included the use of bilingual instructors (*n* = 6) in 30% of the interventions [18, 33–35, 39, 45]. Self-management strategies including goal-setting and problem solving were used in 30% of the interventions (*n* = 6) [31, 32, 34, 37, 40, 45]. Behavioral counseling was also used as a behavior change strategy (*n* = 4) [30, 33–35], with 15% (*n* = 3) [34, 42, 44] of the interventions building in counseling via telephone. In addition, the use of personalized programs and individualized programs was incorporated into 15% (*n* = 3) [31, 32, 41] of the interventions.

### 4.5. Intervention Outcomes and Measures

For 90% of the interventions (*n* = 18), behavior change related to physical activity was measured, whether it was by self-report via logs or checklists (*n* = 9) [3, 31, 35, 37, 38, 41, 42, 44, 45], a 7-day recall (*n* = 6) [18, 25, 28, 32, 34, 39], or through the use of pedometers (*n* = 1) [3] or accelerometers (*n* = 2) [30, 42]. Body mass index (BMI) was measured in 55% (*n* = 11) of the interventions [3, 27, 28, 32–37, 39, 45], although it was not necessarily used as the primary outcome measure in each intervention. Other measures included clinical tests related to diabetes and/or cardiovascular disease (*n* = 9) [3, 18, 27, 28, 34, 35, 39, 43, 45], other anthropometric measures (*n* = 6) [27, 35, 39, 41, 44, 45], social support questionnaires (*n* = 6) [28, 32, 34, 39, 41, 44], measures of acculturation (*n* = 2) [28, 34], stage of change/motivation (*n* = 4) [33, 34, 41, 43], fitness testing (*n* = 4) [18, 27, 30, 45], physical activity attitudes/knowledge/awareness (*n* = 4) [34, 36, 44, 45], self-efficacy for physical activity (*n* = 2) [34, 44], and psychological well-being (*n* = 2) [3, 37].

It is important to recognize that 95% (*n* = 19) of the interventions reported success in some manner (i.e., change in physical activity, knowledge, fitness, etc.). Of those measuring physical activity as an outcome, 72% (*n* = 13) indicated an improvement.[3, 18, 25, 30–32, 34, 35, 37, 41, 42, 44, 45] Five interventions reported an increase in minutes walking and/or associated METS [18, 34, 37, 42, 44]. Three interventions reported an increase in individuals meeting recommended physical activity levels [3, 31, 41]. Two interventions indicated an increase in MVPA [32, 35] and one an increase in VPA [18].

As indicated earlier, there were other measures of success for the interventions. Four of the interventions reported an improvement in either physical activity knowledge or awareness [32, 34, 36, 45]. Social support reportedly increased in 83% (*n* = 5) of the interventions which measured it in some capacity [18, 28, 29, 34, 44]. Improved psychological well-being including decreased stress and depressive symptoms was reported in two of the interventions [3, 37]. Fitness assessments improved in three of the interventions [18, 40, 45]. Clinical measures related to diabetes and/or cardiovascular disease also reportedly improved in three of the interventions [25, 33, 35]. Self-efficacy for physical activity had significant improvements in two of the interventions [29, 45]. Finally, two of the interventions reported a significant decrease in BMI at followup [28, 45]. Yet, it is important to note that only 25% (*n* = 5) of the interventions conducted a follow-up measure; two at 2 months [31, 34], one at 6 months [36], and two at 12 months [18, 33]. Sustainability of behavior change related to physical activity outcomes was not indicated among these five interventions.

## 5. Discussion

The purpose of this systematic review was to summarize the existing evidence related to physical activity interventions with the goal of obesity prevention in Hispanic adults, published between January 1988 and April 2011, in order to determine the benefits and limitations of selected strategies. Based on a review of the resulting 20 interventions, it is evident there is a need for more interventions that specifically target high-risk ethnic populations, including Hispanics. This paper was limited to interventions in which the population included more than 35% Hispanic adults; however, almost half of the interventions were comprised of 100% Hispanic individuals, and 80% of the interventions included over 70% Hispanics among the target population. This was similar to results found in a review (of lifestyle behaviors) by Villarruel and colleagues in 2007 in which 82% of the adult interventions reported samples comprised of 75%–100% Hispanics [26]. There is a need for future interventions which specifically target the Hispanic population, as a homogenous makeup of participants increases the capability of addressing specific language and cultural needs of the population [36].

Half of the interventions targeted females specifically, which makes sense due to the increased prevalence of obesity among Hispanic women [6]. Furthermore, over 70% of Hispanic women report that they do not participate in leisure time physical activity [14], thereby increasing the risk for overweight and obesity. In addition, interventions recruited specific populations including low income [3, 18, 30, 32, 33, 35], sedentary [18, 40, 43], obese [28, 32, 39], those with diabetes [3, 27, 29], and individuals at risk for cardiovascular disease [33]. Targeting these subgroups is important considering physical activity that is an essential component of diabetic care, associated with a decline in cardiovascular disease risk [3], and directly linked to the prevalence of obesity [46]. In fact, based on their results, Castaneda and colleagues suggest that adopting a more physically active lifestyle has potential to reduce diabetes medication by patients and deserves further investigation [27]. Consequently, it is important to consider that these interventions were primarily designed for their target population, and thus results may not be generalizable to all subgroups of the Hispanic population.

Community-based interventions were the most prevalent, which makes sense when targeting the Hispanic population, as it has been reported that a sense of community and social support are extremely important within their culture [11]. Considerations in working with this population include finding innovative, community-based strategies to recruit and retain individuals [34]. Incorporating community collaboration into the interventions improved the reach of the program and showed community buy-in, which essentially built trust and rapport with the participants [31]. Olvera and colleagues structured their community-based intervention to take advantage of the already existing community resources [30]. Similarly, Leeman-Castillo and colleagues gave individuals referrals for local community resources to support their goal(s) and said the support of the community was integral to the success of such an approach [31]. Yan and colleagues also indicated the importance of community agencies in recruitment and implementation of such programs [40]. Using community centers, parks, playgrounds, grocery stores, and schools engaged the participant, and set the program up for sustainability, which tends to be difficult with many physical activity programs, particularly among Hispanic individuals [30]. Of the 14 interventions which were community-based, all achieved positive outcomes but one [39]. Specifically, 78.6% of the community-based interventions improved physical activity [3, 18, 31, 32, 34, 35, 37, 41, 42, 44, 45].

Due to the varying nature of the intervention setting, the duration of each intervention, the target population, the theoretical frameworks used, and the strategies used for each intervention were extremely different. Ingrained into 65% of the interventions was the idea of social support. Within the Hispanic population, social support is described as having a friend who is supportive [18], knowing people who exercise or seeing people exercise [19], or being involved in a group exercise activity [20]. Interventions which built on the assumption that social support has been identified as a strong motivator of physical activity [3, 11, 17] found success, as 60% resulted in an increase in physical activity levels.

 Keller and Cantue encouraged a planned walking group among a group of women. They found the women became *comadres* and provided each other with encouragement and support [28]. Martyn-Nemeth and colleagues uncovered similar trends and found social support increased the likelihood of participation in physical activity; an unintended benefit was the friendships which were formed throughout the duration of the intervention [3]. Another intervention also promoted walking; using groups led by individuals who had already demonstrated success and expressed a desire to help others found success [29]. Building on the potential impact of social support, combined with the importance the Hispanic community places on family, one intervention took advantage of natural interactions occurring in Latino mother-daughter pairs [30]. This unique relationship would be beneficial to improve physical activity among Hispanic women. Participants reported improved social support from family and friends initially after the intervention. However, at followup, Castro and colleagues found this decreased significantly [44]. When the intervention ended, the lack of contact with the counselor and potential loss of interest from family and friends may relate to the decrease in social support. This must be considered when designing future interventions, that is, how can the sense of social support be maintained?

When targeting underserved groups, it is necessary to incorporate individually tailored programs and those which participants find fun and enjoyable, to increase likelihood of action. Keele-Smith found that participants in the group receiving individually tailored exercise prescription were more likely to be consistent exercisers [41]. Motives behind individual choices and behaviors are important to consider when designing program, and seem to improve and/or maintain compliance, particularly with this ethnic population. Utilizing dance was considered an enjoyable, safe, and low-cost method to promote physical activity among Hispanic participants [3]. It was also found that a culturally tailored aerobic dance program increased vigorous physical activity, walking, and fitness levels in overweight Hispanic participants [18].

 Culturally appropriate messages were incorporated into 45% of the interventions, including the use of focus groups to assist in the design and implementation and the creation of culturally relevant materials. The goal of Hispanic-specific interventions includes specificity of cultural values and beliefs and addressing widespread barriers among this population [29]. Participants responded favorably when receiving the intervention in Spanish and appreciated information addressing culture-specific barriers to physical activity for Latinos [32].

Interventions which included staff from the same ethnic group of the population reportedly improved recruitment [47]. The reason being that participants may feel more comfortable when they perceive individuals involved are familiar with their culture, and a sense of trust is increased. Community health workers have also been considered an important element of community empowerment strategies to address health disparities [48]. In one intervention, culturally competent community health workers played a key role in minimizing dropout rates, through encouragement and follow-up phone calls [33]. Community health workers were also used as a mean to facilitate understanding of messages previously provided by counselors and/or through health education, and this combination was found to be successful in promoting behavior change [35]. Chen and colleagues reported difficulty in attaining this when working with a diverse ethnic makeup. They said the lack of heterogeneity between counselors and participants could have reduced the effectiveness of their intervention [42]. These strategies must be considered to make the most impact on physical activity behaviors of the Hispanic population.

Theory-based health behavior change programs are thought to be more effective compared to those that do not use theory [49]. Of the included interventions, 75% incorporated the use of theory, with social cognitive theory [30, 32, 35, 39, 42] and the transtheoretical model [32–34, 37, 40] used most frequently. Theory-based programs aid in the development of measurable program outcomes, help in the design of interventions, provide a framework for effective programming strategies, and increase the likelihood of successful replication [50] and should therefore be considered when designing future interventions.

Duration of the interventions ranged from one session to twelve months. For these interventions, duration was not necessarily correlated with more impactful results. However, as recommended by Prochaska and colleagues [51], for sustaining behavior change, interventions should be at least 6 months in duration. Six of the reviewed interventions met that criteria, thus relapse prevention and maintenance efforts must be considered [52]. However, of those six interventions, only one included followup measures at twelve months [18]. Thus, sustainability of behavior change cannot be indicated. Future studies should consider long-term follow-up and incorporate relapse prevention strategies.

 With all of the different primary outcome measures, only limited comparisons can be made across studies; however, results of these 20 interventions were promising. Of the interventions, 72% reported an increase in physical activity levels at postmeasurement. For example, Hovell and colleagues reported more vigorous exercise and walking, and meeting ACSM guidelines increased from 19% to 63% among participants [18]. Another significant increase in physical activity level occurred in the 6-month intervention emphasizing behavioral strategies; moderate-to-vigorous physical activity increased from 16.5 minutes per week to 147.3 minutes/week [32]. Two interventions reported a reduction in BMI, although significant findings were only found in the study which was 9 months in duration [28]. Social support increased the likelihood of overall participation in two of the interventions [28, 29], again reinforcing the importance of social support in this population. Results also showed improved psychological well-being [3], as well as, improved physical activity knowledge and awareness [36]. These varying measures of success are promising and showcase the feasibility and potential for physical activity interventions among the Hispanic population.

### 5.1. Limitations

It is important to note the limitations of this paper. This is a narrative review and not a quantitative meta analysis. Hence, comment on aspects such as effect sizes for all studies, correlation coefficients, and other quantitative measures cannot be made. Although various subsets of questions exist within this literature, the paper purpose was framed in a manner that was not so prespecified, allowing for a more iterative method of review. Further, the interventions included were limited to those in the English language, published between January 1988 and April 2011 and the location of study must have been in the United States. This precluded interventions from other countries which may have also targeted Hispanic adults. The rationale behind this was that many of the environmental influences Hispanic adults deal with who live in the United States vary compared to those living in other countries.

### 5.2. Recommendations for Improving Interventions

The success of the summarized physical activity interventions among Hispanic adults is quite promising considering the implications this has for future obesity prevention and other chronic disease prevention efforts. Even though there were varying types of interventions, lessons can be learned from those which are found to be the most successful. When designing an intervention for any population, it is important to consider what strategies will be most effective in increasing the likelihood of participation. This is particularly important with a population which might be less likely to follow-through with such programs. Typically, participation rates decrease significantly with the Hispanic population [30]. This necessitates the importance of choosing activities that are appealing and fun, as well as, culturally relevant [18]. Walking programs can increase physical activity among socioeconomically disadvantaged groups; yet they are low-cost, culturally appropriate, and community based [37], all of which tie into those strategies found to be most effective in the Hispanic population.

Interventions among Hispanic populations should build on their sense of culture and incorporate means of social support to increase physical activity levels [29]. Sense of commitment, self-efficacy, and a strong sense of group identity and cohesion were all important factors in Hispanic adults participating in the physical activity interventions [29]. Building in educational opportunities as well as the ability for participants to enhance self-management skills resulted in participants being more likely to achieve positive benefits, particularly those related to an increase in physical activity levels.

 Much of the improvement in the benefits achieved during the interventions tends to be lost in follow-up measures. As a result, there is a need to continue support once the intervention is complete; changes in community-wide policies may include supportive social norms and community-based resources [18]. With such a focus on individual-level variables as primary outcomes, these do not necessarily provide a full understanding of the potential for long-term community mobilization and change. Castro and colleagues recognize the complexity in changing physical activity behaviors, particularly in previously sedentary individuals. They acknowledge that future studies must consider environmental factors and other social and cognitive factors such as competing demands, stressful life events, role models, for physical activity, and normative and cultural beliefs about the importance of physical activity [44]. Evaluation of community level indicators is needed to demonstrate changes in community capacity, resource identification, and environmental change [53].

## 6. Conclusion

Considering the lack of physical activity among Hispanic American, and the associated barriers, the institute of medicine has expressed an urgent need to initiate interventions which influence obesity-related behaviors among diverse ethnic groups [24]. The lack of research in this area is of particular concern since the issue of lifestyle behaviors is likely to become a more prominent public health concern, as the Hispanic population continues to increase in the United States [26]. Greater attention needs to be given to the needs of such underserved populations [13].

Although it is critical to impart individual level behavior change, it is also crucial to address broader policy and environmental-level changes, as to impact individuals and communities. In fact, Healthy People 2020 recommends increasing legislative policies for the built environment that enhance access to and availability of physical activity opportunities [4]. Future interventions must attempt to combine such health promotion efforts to have a greater impact [25].

## Figures and Tables

**Table 1:** Summary of Physical Activity Interventions in Hispanic Adults.

Study	Age/% of Hispanic participants	Theory	Design & sample	Measures	Intervention	Duration	Salient findings
Albright et al. [34]; Collins et al. [54]	18–66 years; 70% Mexican American/Latino women	Transtheoretical Model	Two-group-repeated measures RCT; *N* = 72	Knowledge; perceived barriers to exercise; self-efficacy for PA; social support for exercise; motivational readiness for PA; processes of change; decisional balance; 7-day PA recall; acculturation; BMI; CVD risk factors	Eight 1-hour weekly behavioral skill building sessions; focused on overcoming barriers, setting short-term goals, and developing a PA program; cultural tailored curriculum including ethnically matched health educators; home-based randomized trial began after the series of classes and included either mail support or ongoing PA counseling via telephone and mail (14 calls over 10 months)	8 weeks then 10 months	After preintervention 8-week preparatory course, there was a significant increase in knowledge, perceived social support, walking minutes per week, and total cognitive and behavioral processes (*P* < .01). After 10 months of a home-based intervention, women in the phone + mail counseling condition had a significantly greater increase in estimated total energy expenditure compared to women in the support condition (*P* < .05)

Avila & Hovell [45]	40–44 years	Not mentioned	Two-group-repeated measures RCT; *N* = 44	Attitudes; beliefs; knowledge of exercise; MVPA; BMI; BP; glucose; cholesterol; waist/hip circumference; 1-mile walk and estimated VO_2_ max	Eight 1-hour sessions consisting of self-change behavioral modification; assistance from an assigned buddy (social support); stretching and walking component (led for 20 mins. of walking during each session) conducted by bicultural Spanish speaking physician	8 weeks	Statistically significant (*P* < .05) decrease compared to control group for BMI, waist-to-hip ratio, and cholesterol; significant increases (*P* < .05) for VO_2_ max, exercise rate frequency, self-efficacy, fitness level, and knowledge

Bopp et al. [36]	Mean age = 42.5 years (SD = 12.1); 81.1% of Mexican descent	Not mentioned	Three-group RCT (2 intervention, 1 comparison); *N* = 50	Process evaluation outcomes; PA knowledge; height; weight; program barriers; activity awareness	Faithful Footsteps Program; Faith-based physical activity intervention; culturally and spiritually relevant educational materials and activities developed promoting the health benefits of PA; team-based walking contest to promote social support for PA; health “fiesta” provided hands-on educational opportunities for PA	8 week	66% of participants identified health reasons for participating in PA (compared to 36%); 47% accurately described PA recommendations (versus 16%)

Castenada et al. [27]	Mean age = 66 years; all Hispanic, Caribbean descent (84–90%)	Not mentioned	Two-group-repeated measures RCT; over 55 years; type 2 diabetes; *N* = 62	Glycemic and metabolic control; BMI; WHR; % body fat; 7-day PA recall; muscle strength with 1RM	Structured 45 mins. exercise session 3 times/week; progressively increased intensity	16 weeks	Leisure and household physical activity levels significantly improved in intervention group *P* < .001); improved glycemic control, decreased diabetes medications

Castro et al. [44]	24–55 years; 45.3% Hispanic	Self-management model	Two-group-repeated measures RCT; *N* = 53	PA minutes per week; barriers, enjoyment; self-efficacy; social support	Walking program with one session per week; participants given written materials and health and weekly phone counseling sessions; focusing on informational control, education, social support, motivation, problem-solving, and improving self-efficacy	6 weeks	At 5-month followup, PA, barriers, enjoyment, and self-efficacy were not significant; increase in social support was significant (*P* < .01); both conditions increased walking minutes per week (*P* < .001)

Chen et al. [42]	23–54 years; 44.5% Hispanic women	Social cognitive theory	Two group by three repeated measures quasiexperimental design (randomized to comparison or treatment); *N* = 128	Self-reported walking; subsample-used accelerometers	Home-based behavioral intervention to promote walking; intervention group received six phone calls (20–30 mins.) with counseling versus educational phone calls intended to increase self-efficacy, assess barriers, problem solve to promote social support	8 weeks	Both conditions increased self-reported walking at the 2 months after test(*P* < .88), with mean change of 86 and 81 mins./week for behavioral and educational group, respectively

Grass et al. [38]	18–55 years; 72% Hispanic women	Not mentioned	Nonexperimental-repeated measures design; *N* = 130	PA minutes per week; PA barriers	Participatory action research; four sessions over 3 months of “walking clubs”; family focused to influence social support; written materials in English and Spanish	3 months	No significance in PA; PA barriers significance (*P* < .05)

Hayashi et al. [33]	40–64 years; 100% Hispanic Women	Transtheoretical model	RCT at 4 sites; lower income under or uninsured; at risk for CVD; *N* = 869	Stage of readiness questionnaire; cholesterol, BP, BMI, coronary heart disease risk; PA level/ intensity/barrier	Wisewoman; delivered by community health workers who were bilingual and bicultural; focused on health behavior counseling	3 lifestyle sessions (30–45 mins.)	Improvement in PA readiness for change in 68% of intervention group; achieving a high degree of improvement in PA was twice as likely; improvement in estimated 10-year CHD risk

Hovell et al. [18]	18–55 years; 100% Hispanic Women	Operant learning theory	Two-group-repeated measures RCT; low income; sedentary immigrants; *N* = 151	Physical activity; aerobic fitness VO_2_ max; height; weight; BP; glucose; insulin; lipid measurements	Three 90-minute sessions per week of supervised aerobic dance in a community setting; 5 : 1 participant to staff ratio; bilingual Aerobic instructor; 30-mins. of exercise/diet education after each session including culturally appropriate materials; problem-solve barriers; assigned exercise buddy	6 months	More vigorous exercise and walking at posttest for intervention group (*P* < .001); meeting ACSM guidelines increased from 19.1% to 63.2% in intervention group compared to control group (13.6% to 16.7%); sig increase in VO_2_ max (*P* < .01)

Ingram et al. [29]	33–95 years; 100% Hispanic	Grounded theory	Qualitative (focus groups); w/diabetes; *N* = 20	Focus group explored themes related to self-efficacy and social support (conducted in Spanish)	Animadora study; community-based intervention to promote walking; series of walking groups led by individuals who had demonstrated success and expressed desire to help others; met 3 times/week	12 weeks	Social support expressed as commitment and companionship; walkers demonstrated a high level of self-efficacy for walking; development of group identity/social cohesion was a motivator to walk

Keele-Smith [41]	18–59 years; staff and students at New Mexico University; 42% Hispanic	Reversal theory	Two-group-repeated measures RCT; *N* = 149	PA frequency and duration; weight, body fat; exercise motivation; social support	Participants given brochure highlighting general information about exercise; individualized-written exercise prescription developed based on baseline data; one-on-one weekly educational seminars 30–45 mins.; monitoring only group that received weekly phone calls	5 weeks	More participants in intervention group were meeting PA recommendations; no significant differences in weight, body fat; consistent exercisers had significantly higher motivation scores than did inconsistent exercisers

Keller and Cantue [28]	45–70 years; 100% Hispanic women	Not Mentioned	Two-group-repeated measures RCT; women who were postmenopausal, obese, and sedentary; *N* = 18	bioelectric impedance and BMI; anthropometric measures; total serum cholesterol; PAR; PA log; community/friend/family assessment for exercise survey; acculturation scale	Camina por Salud; clinical feasibility study designed to evaluate the effects of two frequencies of walking (3 versus 5 days/week); 30 minutes at the pace of a 20-minute mile (3.2-MET intensity	36 weeks	Significiant differences in BMI reduction, (*P* = .001); No significiant difference in anthropometric and blood lipid results; No significiant relationship between the mins. walked/week and acculturation or neighborhood characteristics. For Group I, there was a strong correlation between mins. walked and social support scores (*r* =.99, *P* = .04)

Leeman-Castillo et al. [31]	31–50 years; 100% Hispanic	Social science theory	Nonexperimental two-group-repeated measures design; Spanish & English speaking recruited ≥21 years; *N* = 299	Self-report PA	LUCHAR; Community-based health kiosk program, English or Spanish; users receive personalized feedback from computerized role models that guide them in establishing goals; printout at the completion of the program includes personal program summary and referrals for local resources	1-session; 2-month followup for risk assessment	Significant increase in participants meeting PA recommendations in community setting (33% to 49%) and clinic setting (45% to 65%) at 2-month followup

Martyn-Nemeth et al. [3]	30–65 years; 100% Hispanic	Social ecological model	Nonexperimental one-group-repeated measures design; w/type 2 diabetes; low income; *N* = 16	Hemoglobin A1C, lipids, psychological well-being; BMI; daily exercise log	Community-based, culturally designed exercise program through dance (60 mins.) received weekly exercise appointment cards	12 weeks	80% of the reported becoming physically active at least 6 days per week or more; no significiant change in BMI; trend toward improved psychological well-being & diabetes measures

Mier et al. [37]	Mean age = 32.4 years; 93.8% Mexico country of origin	Transtheoretical model	Nonexperimental one-group-repeated measures design; *N* = 16	Physical walking level; depressive symptoms; stress; BMI	Spanish handbook (Let's Walk) developed to include information which was culturally appropriate used individualized problem-solving and self-management strategies; use of social support	12 weeks	Significant differences for walking MET (*P* < .02); level of depressive symptoms and stress were significantly reduced (*P* < .05)

Olvera et al. [30]	28–48 years; 100% Hispanic mother-daughter pairs	Social cognitive theory	Two-arm experimental design; lower income mother/daughter pairs; *N* = 46 pairs	Acculturation scale; BMI; shuttle run test or rockport walk test; accelerometers; SPAN survey; nonexercise PA rating	Bounce; family-based program delivered in community and school settings; 3-week structured group aerobic, sport sessions, or free play recreational activities; 1-week behavioral counseling session	12 weeks	No significiant differences in mother's physical fitness or PA levels; no significiant differences in BMI; although daughters did exhibit significant changes in physical fitness and PA levels (*P* < .05)

Pekmezi et al. [32]	18–65; 100% Hispanic women	Transtheoretical model; social cognitive theory	Two-group-repeated measures RCT; low-income, acculturated, majority overweight/Obese; inactive; *N* = 93	Self-report PA, 7-day PA recall; height, weight; social support; environmental access scale; CES-D scale; stage of change	Seamos activas; emphasized behavioral strategies such as goal-setting, monitoring, problem-solving, barriers, increasing social support, and rewarding oneself for meeting PA goals; monthly educational materials mailed based on individual-level-tailored feedback	6 months	MVPA increased from 16.56 mins./week to 147.27 min.; significiant increase in cognitive and behavioral processes of change (*P* < .01)

Poston et al. [39]	Mean age = 39.2 years; 70% USA. born Hispanic women	Social cognitive theory	RCT prospective block design (preestablished social groups); overweight or obese; *N* = 269	7-day PAR; BMI; WHR; blood lipids; BP; social support; health locus of control	One session per week for 12 months focused on influence of education, use of social support networks, dealing with negative influences, and restructuring personal environment; instructors were bilingual; bilingual materials; participated in 30 mins. of walking during the weekly meeting and walking clubs set up during the week	12 months	Intervention participants were not more active than controls at 6 or 12 months; no significant changes in BMI, PA recommendations, and blood lipids; significantly fewer participants who met the activity goal in the treatment group compared to wait-list control group at baseline (22% versus 25%)

Staten et al. [35]	Mean age = 57.2 years; 74% Hispanic women	Social cognitive theory	Three-group (interventions) randomized experimental design; uninsured over 50 years; *N* = 217	BMI; WHR; cholesterol; glucose; activity frequency questionnaire	One group received provider counseling (PC) (active control); 2nd group received health education classes and a monthly newsletter as well as PC (PC + HE); 3rd group received all of the above and social support provided by community health workers (PC + HE + CHW); CHW were bilingual Hispanic women; CHW led bimonthly walks and encouraged participants to find walking partners, build social support	12 months	All groups showed significant increase in MVPA with no significant differences between groups; BP decreased significiant among PC + HE + CHW (*P* < .05) and PC + HE; no significiant change in BMI

Yan et al. [40]	Mean age = 72.9 years; 50.5% Hispanic	Transtheoretical model	Quasiexperimental design (intervention and small wait-list comparison); over 50 years; sedentary; *N* = 208	Participation rates; physical performance	Active start: 1 hour per week in a group setting to set goals, identify barriers, and establish social support system; after week 4, participants met 3 times/week for 45 mins.; exercises were performed to culturally preferred music; given safe exercises at home handout	6 months	Significant improvements in fitness testing measures among intervention group, including Hispanics within this group (*P* < .001)
